# Computed Tomography Findings of Pulmonary Lymphoma in a Dog and Two Cats

**DOI:** 10.1002/vms3.70947

**Published:** 2026-04-07

**Authors:** Toshiyuki Tanaka, Yusuke Wada, Takuya Kusaka, Mizuki Tomihari, Takashi Hasegawa

**Affiliations:** ^1^ Laboratory of Veterinary Advanced Diagnosis and Treatment School of Veterinary Science Osaka Metropolitan University Osaka Japan; ^2^ Kinki Animal Medical Training Institute & Veterinary Clinic Osaka Japan; ^3^ Japan Animal Referral Medical Center Osaka Hospital Osaka Japan; ^4^ Veterinary Medical Center School of Veterinary Science Osaka Metropolitan University Osaka Japan

**Keywords:** canine, feline, primary, pulmonary lymphoma, secondary

## Abstract

This case series assessed CT findings of pulmonary lymphoma in a dog and two cats. The dog (Case 1) showed defined consolidation and nodules with enlarged sternal and mediastinal lymph nodes. Air bronchogram and distinct pulmonary vessels were observed within the lesion. Several nodules were also found in the kidneys. Pulmonary and kidney lesions were diagnosed as B cell lymphoma. One cat (Case 2) showed well‐defined consolidation with an enlarged tracheobronchial lymph node. Air bronchogram and distinct pulmonary vessels were also observed in the lesion. No additional lesions were detected. The pulmonary lesion was diagnosed as B cell lymphoma. Another cat (Case 3) showed a nasopharyngeal mass and multiple well‐defined pulmonary nodules without lymphadenomegaly. Pulmonary nodules and the nasopharyngeal mass were diagnosed as lymphoma. In Case 3, T/B classification was not performed. Of three pulmonary lymphoma cases, the distinguishing imaging feature was a well‐defined lesion with distinct air bronchograms, pulmonary vessels and homogeneous enhancement.

## Graphical abstract

This study was retrospective case series analysis of CT findings of pulmonary lymphoma in a dog and two cats. Of three pulmonary lymphoma cases, the distinguishing CT feature was a well‐defined lesion with distinct air bronchograms, pulmonary vessels, and homogeneous enhancement.

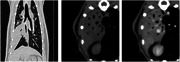

## 1 Introduction

Pulmonary involvement in lymphoma is classified as either primary or secondary, with secondary occurring as part of multicentric or extranodal disease (Vail 2007). Pulmonary involvement is reported in more than 60% of dogs with lymphoma (Geyer et al. 2010; Hawkins et al. 1993), while pulmonary involvement in cats is unusual (Geyer et al. 2010; Gabor et al. 1998; Taylor et al. 2009). Among cats with lymphoma, 12.8% exhibited pulmonary involvement, all of which were classified as secondary pulmonary lymphoma (Leite‐Filho et al. 2018). The mixed anatomical category, in which two or more anatomical forms are involved, is the most common type of lymphoma observed, accounting for 50% of cases (Gabor et al. 1998; Louwerens et al. 2005). Although there are limited reports evaluating pulmonary lymphoma treatment, chemotherapy is frequently administered (McKay et al. 2004; Leach and Van Camp 2010).

In dogs, carcinoma is the most commonly observed pulmonary tumour, followed by sarcoma, adenoma, pulmonary neuroendocrine tumour, plasmacytoma and carcinosarcoma (McPhetridge et al. 2021). In cats, adenocarcinoma is the most common pulmonary tumour, followed by adenosquamous cell carcinoma and undifferentiated squamous cell carcinoma, both typically of bronchial origin (Aarsvold et al. 2015). The decision to pursue surgical resection depends on the suspected tumour type, expected survival time and likely perioperative morbidity and mortality rates (McPhetridge et al. 2021).

CT is an effective tool for preoperative staging of pulmonary tumours, enabling detailed evaluation (Ballegeer et al. 2010; Nunley et al. 2015). In dogs and cats, CT findings of pulmonary tumours including carcinoma, fibrosarcoma, bronchial origin tumour, adenosquamous cell carcinoma, undifferentiated squamous cell carcinoma and eosinophilic pulmonary granulomatosis are reported (Aarsvold et al. 2015; Marolf et al. 2011; Almendros et al. 2022). As pulmonary lesion biopsy or resection is invasive and impractical in many patients (Zekas et al. 2005; Turner et al. 2017), improved tumour differentiation based on CT findings is optimal. Because lymphoma has some treatment options, we believe identifying the CT findings of pulmonary lymphoma is needed. To the best of our knowledge, there are no detailed reports of pulmonary lymphoma CT evaluation in dogs and cats. This study seeks to address this by retrospectively assessing the CT findings of pulmonary lymphoma in dogs and cats.

## 2 Methods

This study was conducted as a retrospective case series analysis. Owners of the animals described in this study provided informed consent for the diagnostic procedures, treatment and use of clinical data (such as medical history, imaging studies and histopathological findings) for research and publication purposes. As all diagnostic studies and treatments were part of daily clinical activities, the threshold for submission to the local ethics and welfare committee was not reached.

All animals suspected of pulmonary lesions that underwent CT examinations at our institution between 2015 and 2024 were considered for inclusion in the study. Inclusion criterion was histopathological confirmation of the pulmonary lesion as lymphoma. Animals with multiple concurrent neoplasms were excluded. Animals were selected for inclusion or exclusion by a veterinarian with 19 years of clinical experience. A dog (Case 1) and two cats (Case 2 and 3) with histologically confirmed lymphoma that underwent whole‐body CT examination were identified. For the CT examination, the animals were anaesthetized with intravenous propofol (propofol 1%; MSD Animal Health K.K., Tokyo, Japan) and maintained with isoflurane and oxygen. All animals were placed under general anaesthesia and placed in the supine or prone position. CT examinations were performed using a SOMATOM Scope (Siemens AG, Munich, Germany) or Activion 16 (Canon Medical Systems Corporation, Tochigi, Japan). CT acquisition settings of the SOMATOM Scope and Activion 16 were standardized according to our previous protocol (Tanaka et al. 2019).

For contrast‐enhanced imaging, all animals were administered 2 mL/kg (300 mgI/mL) of the non‐ionic contrast medium iohexol (Ioverin 300; Teva Pharma Japan, Inc., Aichi, Japan) via an indwelling intravenous catheter placed in the cephalic vein. The injection duration was 20 s. A contrast‐enhanced study was performed in the arterial (20 s after the injection of contrast medium), portal (60 s) and equilibrium phases (180 s). Images were displayed in an abdominal window setting (window level = 35 Hounsfield units [HU], window width = 360 HU) and pulmonary window setting (window level = −600 HU, window width = 1500 HU) to assess pulmonary lesions.

In Cases 1 and 2, contrast enhancement was taken in three phases at the chest. Contrast enhancement of other parts of the body was performed in only the portal phase. In Case 3, contrast enhancement was taken in three phases at the head. Contrast enhancement of other parts (including thorax and abdomen) were only performed in the portal phase.

Image analyses were performed using a commercially available DICOM image viewing software (OsiriX 13.0.1, 64 bit, Pixmeo, Switzerland). All CT images were reviewed by two veterinarians, each with at least 10 years of specialty veterinary radiology experience, with CT features recorded by consensus. Observers were aware of final diagnoses at the time of CT image review. Images were assessed in random order three times with at least a 2‐week interval to avoid potential bias; mean CT values were then calculated.

The following qualitative CT features were recorded: shape of pulmonary lesion (consolidation or nodule), pulmonary lesion area involved, other involved organs, lesion enhancement pattern in each post‐contrast phase (homogeneous or heterogeneous), lymphadenomegaly (presence or absence) and location of lymphadenomegaly.

Consolidation is defined as a non‐nodular area of increased attenuation that obscures underlying vessels on pre‐contrast CT with a pulmonary filter (Wislez et al. 1999). A nodule was defined as an oval‐shaped lesion with a diameter of less than 3 cm (Leite‐Filho et al. 2018). The enhancement pattern in each phase was considered homogeneous or heterogeneous, depending upon whether there was less than or more than 10 HU difference in enhancement (Zhu et al. 2012). Attenuation, in HU, was measured on pre‐contrast and post‐contrast images by manually selecting three regions of interest (ROI) to fit the lesion, excluding vessels and bronchi. Lymphadenomegaly was defined as lymph node length, width or thickness exceeding the mean dimensions of normal lymph nodes (Milovancev et al. 2017; Smith et al. 2020; Kayanuma et al. 2020).

## 3 Results

### 3.1 Case 1 (Canine Case)

A 10‐year‐old female Chihuahua was presented due to an incidentally detected chest mass without clinical sign. Clinical examination was normal, as were haematological and serum biochemical analyses. CT examination revealed well‐defined areas of consolidation and nodules located in the basal or peripheral regions of the right cranial, right caudal and left caudal lobes. An air bronchogram and distinct pulmonary vessels were observed within the lesion, with the lesion also demonstrating homogeneous contrast enhancement. Lymphadenomegaly was identified in sternal and mediastinal lymph nodes. Several nodules were also found in the kidneys. Pulmonary and kidney lesions were diagnosed as B cell lymphoma by fine needle aspiration (FNA) and clonality testing.

### 3.2 Case 2 (Feline Case)

A 7‐year‐old neutered female mixed‐breed cat was presented with respiratory distress and cough. On radiography, a thoracic mass was detected. Despite 2 weeks of antibiotic treatment, the appearance of the mass remained unchanged. Clinical examination was normal, as were haematological and serum biochemical analyses. CT examination revealed well‐defined consolidation involving the entire right middle, right caudal and right accessory lung lobes. Air bronchograms and distinct pulmonary vessels were observed within the lesion. This lesion also showed homogeneous enhancement. Lymphadenomegaly was detected in tracheobronchial lymph nodes. No other lesions were detected. The pulmonary lesion was diagnosed as B cell lymphoma by FNA and clonality testing.

### 3.3 Case 3 (Feline Case)

A 5‐year‐old neutered female mixed‐breed cat was presented with respiratory distress and anorexia, also testing positive for feline immunodeficiency virus. On radiography, a chest mass was detected. Clinical examination was normal, as were haematological and serum biochemical analyses. CT examination revealed a nasopharyngeal mass and multiple well‐defined pulmonary nodules located in the basal regions of the right middle and right caudal lung lobes. The pulmonary nodules showed homogeneous enhancement. Lymphadenomegaly was not detected. Pulmonary nodules and nasopharyngeal mass were diagnosed as lymphoma by FNA.

**FIGURE 1:**
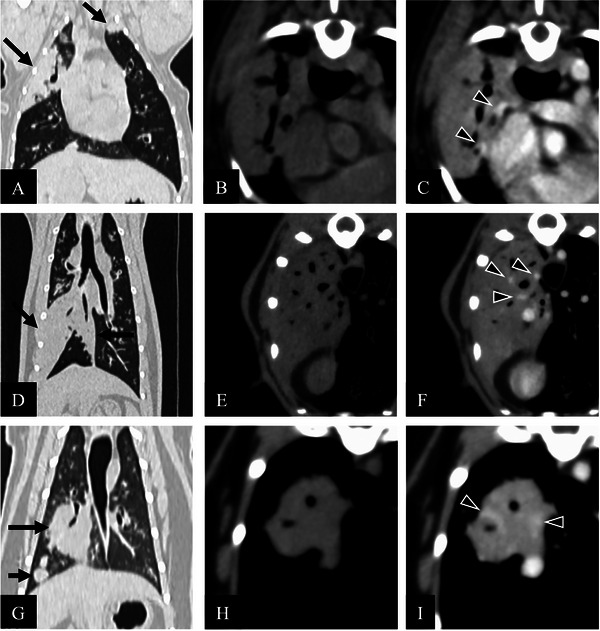
Representative CT images of pulmonary lymphoma including (A–C) Case 1, (D–F) Case 2 and (G–I) Case 3. Images in (A, D, G) multiplanar reconstruction, (B, E, H) transverse pre‐contrast and (C, F, I) transverse portal‐phase post‐contrast phases. (A, black arrows) Case 1 revealed well‐defined consolidation and nodules, (D, black arrows) Case 2 revealed well‐defined consolidation and (G, black arrows) Case 3 revealed nodules. (C, F, I) The consolidation and large nodule indicated distinct air bronchogram, pulmonary vessels (arrowheads) and homogeneous enhancement.

Representative figures for these three cases are shown in Figure 1. CT findings and clinical information of the three cases are summarized in Table 1.

**TABLE 1:** CT findings and clinical information of pulmonary lymphoma.

Case	Animal (breed)	Sex	Age (year)	BT	Diagnosis	Clinical sign	Lesion shape	Feature
1	Dog (Chihuahua)	F	10	WNL	B lymphoma	None.	Consolidation. Nodule.	Air bronchograms. Pulmonary vessels.
2	Cat (mix)	SF	7	WNL	B lymphoma	Respiratory distress. Cough.	Consolidation.	Air bronchograms. Pulmonary vessels.
3	Cat (mix)	SF	5	WNL	Lymphoma^a^	Respiratory distress. Anorexia.	Nodule.	Air bronchograms. Pulmonary vessels.

CE arterial	CE portal	CE equilibrium	Lymphadenomegaly	Other lesions	Involved area
Homo	Homo	Homo	Sternal mediastinal	Kidney	Basal or peripheral part of right cranial, right caudal and left caudal lobes.
Homo	Homo	Homo	Tracheobronchial	None	Entire right middle lobe. Entire right caudal lobe. Right accessory lobe.
N/A	Homo	N/A	None	Nasopharynx	Basal part of right middle lobe. Right caudal lobe.

Abbreviations: BT, blood test; CE, contrast enhancement; F, female; SF, spayed female; WNL, within normal limits.

^a^ T/B classification not performed.

## 4 Discussion

Characteristic CT findings of pulmonary lymphoma in this study included a well‐defined lesion with distinct air bronchograms, clearly visualized pulmonary vessels and homogeneous contrast enhancement. In humans, primary pulmonary lymphoma typically shows a peribronchial and perivascular distribution histologically (Geyer et al. 2010), with consolidation of homogeneous attenuation, air bronchograms and vascular signs as characteristic imaging features (Cozzi et al. 2019). However, there are no reports evaluating histopathological lymphoma distribution in dogs and cats with primary pulmonary lymphoma. In cats with secondary pulmonary lymphoma, neoplastic lymphocytes are predominantly distributed in a peribronchial and perivascular pattern, followed by pleural, interstitial, nodular and alveolar distributions (Leite‐Filho et al. 2018; Brown et al. 2011). Peribronchial and perivascular infiltration are observed in large granular lymphocyte lymphoma (Darbès et al. 1998). CT findings, such as distinct air bronchograms and pulmonary vessels, may reflect neoplastic lymphocyte distribution. Primary pulmonary tumours in dogs and cats cause bronchial compression or invasion, leading to varying degrees of bronchial narrowing (Aarsvold et al. 2015; Marolf et al. 2011). Gas‐containing cavities are identified in feline tumours of bronchial origin (Aarsvold et al. 2015). Most primary pulmonary tumours have mild to moderate heterogeneous contrast enhancement (Marolf et al. 2011). In the cases presented, the degree of consolidation and presence of large nodules were associated with distinct air bronchograms. Thus, the presence of clear air bronchograms, distinct pulmonary vessels and homogeneous enhancement may assist in identifying pulmonary lymphoma.

At the time of diagnosis for Case 2, only pulmonary lesions and enlargement of tracheobronchial lymph nodes were observed, suggesting primary pulmonary lymphoma. Human primary pulmonary lymphoma is distinguished from secondary pulmonary involvement of systemic lymphoma by the absence of extrapulmonary disease at the time of diagnosis and no detection in other organs, even after 3 months (Sanguedolce et al. 2021; Freeman et al. 1972). Unfortunately, 3‐month follow‐up was not performed in Case 2. Therefore, confirming a diagnosis of primary pulmonary lymphoma was not possible. In Cases 1 and 3, lesions were observed in multiple organs at the time of diagnosis, suggesting secondary pulmonary lymphoma. Pulmonary lymphoma resulted in consolidation and/or nodule formation in these cases. Canine and feline pulmonary lymphoma exhibits variable radiographic appearances, ranging from normal findings to alveolar and/or unstructured interstitial infiltrates, nodules and/or masses and bronchial infiltrates (Geyer et al. 2010). Based on T/B classification, B cell lymphoma typically shows multiple pulmonary mass lesions (Wilson 2017). Unfortunately, Case 3 was not differentiated as to B‐ or T‐cell origin. CT findings of human primary pulmonary lymphoma indicate one or more well‐defined nodules or masses, often with an air bronchogram inside the lesion (Wislez et al. 1999; Cordier et al. 1993). Nodules were also observed in this study. However, air bronchograms were not identified within the small nodules. Further studies are required to evaluate differences in CT findings between primary/secondary and B/T cell lymphomas in a larger cohort of dogs and cats.

CT findings of human pulmonary lymphoma correlate with gross pathological appearance and were related to a neoplastic lymphocyte distribution (Wislez et al. 1999). However, 56.2% of feline pulmonary lymphomas do not show gross abnormalities (Leite‐Filho et al. 2018). Therefore, pulmonary lymphoma may extend beyond the abnormal findings detected on CT examination. Unfortunately, our cases were diagnosed by FNA; therefore, the extent of neoplastic lymphocyte infiltration beyond the lesion detected by CT is unclear.

Image interpretation can be subjective and greatly influenced by education and experience (Bouhali et al. 2022). In humans, along with the computer application technology progress, machine learning have been applied for various fields of medical image analysis, including breast cancer detection, tumour screening, lung nodule classification and prediction of the treatment outcomes (Yin et al. 2024; Zhao et al. 2023). The advantage of deep learning lies in its ability to bypass complex feature extraction steps, directly simplifying the original multi‐stage detection process into an end‐to‐end network (Laghari et al. 2023). In cancer precision medicine, CT features and machine learning are used as paradigm shifts in complementing cancer experts (Munir et al. 2024). In veterinary medicine as well, there is a growing interest in machine learning (Shaker et al. 2021; Currie and Rohren 2022; Currie et al. 2023). Identifying the CT findings of pulmonary lymphoma with a larger sample size may enable future diagnosis and prediction of treatment outcomes using machine learning.

In conclusion, CT findings in canine and feline pulmonary lymphomas include distinct air bronchograms, pulmonary vessels and homogeneous enhancement.

## Author Contributions

T.T. is the principal investigator and first author of this manuscript. T.T. conceived the study and supervised the surveillance components. T.T., Y.W., T.K., M.T. and T.H. validated, analysed and interpreted the data. T.T. prepared the initial draft, figure and table. All authors contributed to the writing and editing of the manuscript.

## Funding

The authors have nothing to report.

## Ethics Statement

The owners of the animals described in this study provided informed consent for the diagnostic procedures, treatment and use of clinical data such as medical history, imaging studies and histopathological findings for research and publication purposes. Since all diagnostic studies and initiated treatments were part of daily clinical activities, the study did not reach the threshold for submission to the local ethics and welfare committee.

## Conflicts of Interest

The authors declare no conflicts of interest.

## Data Availability

Data supporting the findings of this study are available from the first author (T.T.) upon reasonable request.
